# Progression free survival of myeloma patients who become IFE-negative correlates with the detection of residual monoclonal free light chain (FLC) by mass spectrometry

**DOI:** 10.1038/s41408-024-00995-y

**Published:** 2024-03-18

**Authors:** H. V. Giles, M. T. Drayson, B. Kishore, C. Pawlyn, M. Kaiser, G. Cook, R. de Tute, R. G. Owen, D. Cairns, T. Menzies, F. E. Davies, G. J. Morgan, G. Pratt, G. H. Jackson

**Affiliations:** 1 University Hospitals Birmingham NHS Foundation Trust, Birmingham, UK; 2 University of Birmingham, Birmingham, UK; 3 The Institute of Cancer Research, London and The Royal Marsden Hospital, London, UK; 4 Leeds Cancer Research UK Institute of Clinical Trials Research, University of Leeds, Leeds, UK; 5 Haematological Malignancy Diagnostic Service, Leeds Teaching Hospitals Trust, Leeds, UK; 6 Myeloma Research Program, Perlmutter Cancer, NYU Langone Health, New York, USA; 7 Department of Haematology, University of Newcastle, Newcastle upon Tyne, UK

**Keywords:** Myeloma, Myeloma, Prognosis

## Abstract

Deeper responses are associated with improved survival in patients being treated for myeloma. However, the sensitivity of the current blood-based assays is limited. Historical studies suggested that normalisation of the serum free light chain (FLC) ratio in patients who were negative by immunofixation electrophoresis (IFE) was associated with improved outcomes. However, recently this has been called into question. Mass spectrometry (MS)-based FLC assessments may offer a superior methodology for the detection of monoclonal FLC due to greater sensitivity. To test this hypothesis, all available samples from patients who were IFE negative after treatment with carfilzomib and lenalidomide-based induction and autologous stem cell transplantation (ASCT) in the Myeloma XI trial underwent FLC-MS testing. FLC-MS response assessments from post-induction, day+100 post-ASCT and six months post-maintenance randomisation were compared to serum FLC assay results. Almost 40% of patients had discordant results and 28.7% of patients with a normal FLC ratio had residual monoclonal FLC detectable by FLC-MS. FLC-MS positivity was associated with reduced progression-free survival (PFS) but an abnormal FLC ratio was not. This study demonstrates that FLC-MS provides a superior methodology for the detection of residual monoclonal FLC with FLC-MS positivity identifying IFE-negative patients who are at higher risk of early progression.

## Introduction

Serum FLC assays have provided a valuable addition to the diagnostic armamentarium for the detection and monitoring of patients with plasma cell dyscrasias. Their improved sensitivity in comparison to serum IFE has enabled many patients with oligo-secretory and light chain-only multiple myeloma to be monitored using a simple blood test [1–3] and serum FLC responses form an integral part of haematological response assessments in AL amyloidosis. Normalisation of the serum FLC ratio was incorporated into the uniform response criteria for multiple myeloma in 2006 with the addition of the stringent complete response (sCR) category in an effort to identify patients with no residual detectable monoclonal protein using electrophoretic techniques who had improved prognosis [4]. However, since the introduction of this response category conflicting reports have been published regarding the prognostic utility of serum FLC ratio normalisation in multiple myeloma patients who have achieved IFE negativity [5–9].

The discrepant results regarding the prognostic significance of normalisation of the serum FLC ratio in patients who have achieved IFE negativity may at least in part be due to methodological differences between studies, including the way in which an abnormal serum FLC ratio was defined. However, as serum FLC assays indirectly detect the presence of monoclonal FLC based on skewing of the FLC ratio, low-level false positive results may be obtained due to non-disease related-factors, including oligoclonal bands [10] and treatment-related immune suppression [3, 11]. Conversely, as minimal residual disease (MRD) studies have shown that many patients in complete response (CR) still have low-level detectable disease in the bone marrow [5, 12, 13], low-level monoclonal FLC are likely to be present in some patients with normal serum FLC ratios. Deep responses and oligoclonal bands are frequently observed in patients treated with modern treatment regimens [10, 14, 15], so it is unclear whether the serum FLC ratio is still able to provide additive prognostic information in patients achieving IFE negativity.

MS assays are emerging as a potentially more sensitive methodology for monitoring monoclonal proteins compared to the standard electrophoretic assays [16–19]. These assays identify monoclonal proteins based on the mass-to-charge ratio (*m/z*) or amino acid sequence of the monoclonal protein, which allows the monoclonal FLC to be monitored throughout treatment and obviates the need to rely on indirect measures such as the FLC ratio to determine the presence or absence of residual monoclonal FLC. Intact light chain MS assays using FLC specific reagents using matrix-assisted laser desorption ionisation time-of-flight (MALDI-TOF) MS have been shown to provide greater sensitivity for the detection of low-level monoclonal FLC in patients with AL amyloidosis and non-measurable multiple myeloma [20, 21].

FLC-MS assays are distinct from both of the five bead MS assays that have been developed (MASS-FIX by the Mayo clinic and EXENT by The Binding Site Ltd), which only include antisera for total kappa and total lambda light chains (i.e. FLC and light chain that is bound to heavy chain). Long immunoglobulin half-life has been shown to impact detection rates using five-bead assays such as MASS-FIX [18], however this will not be an issue for FLC-MS as the half-life of FLC is only a few hours. This makes exploring more sensitive ways to measure monoclonal FLC an attractive option for the detection of low-level residual disease within the first 3–12 months post-transplant.

To explore this, we compared the sensitivity and prognostic utility of serum FLC and FLC-MS assessments in transplant-eligible patients with newly diagnosed multiple myeloma treated with carfilzomib, cyclophosphamide, lenalidomide and dexamethasone (KCRD) in the Myeloma XI trial who had no residual monoclonal protein detectable by IFE. The results of this testing were then compared to the FLC ratio results obtained using Freelite.

## Methods

### Patients included in this study

This study was an ancillary study utilising residual serum samples from patients who underwent treatment with a minimum of four cycles of induction chemotherapy with KCRD, followed by high-dose melphalan-conditioned autologous stem cell transplantation (ASCT) in the Myeloma XI trial. Post-ASCT patients were randomised 2:1 between continuous lenalidomide maintenance until progression versus observation. All patients provided written informed consent for participation in the trial and ancillary studies. The trial is registered with the EU Clinical Trials Register (2009-010956-93) and the ISRCTN registry (ISRCTN 49407852).

**Fig. 1:**
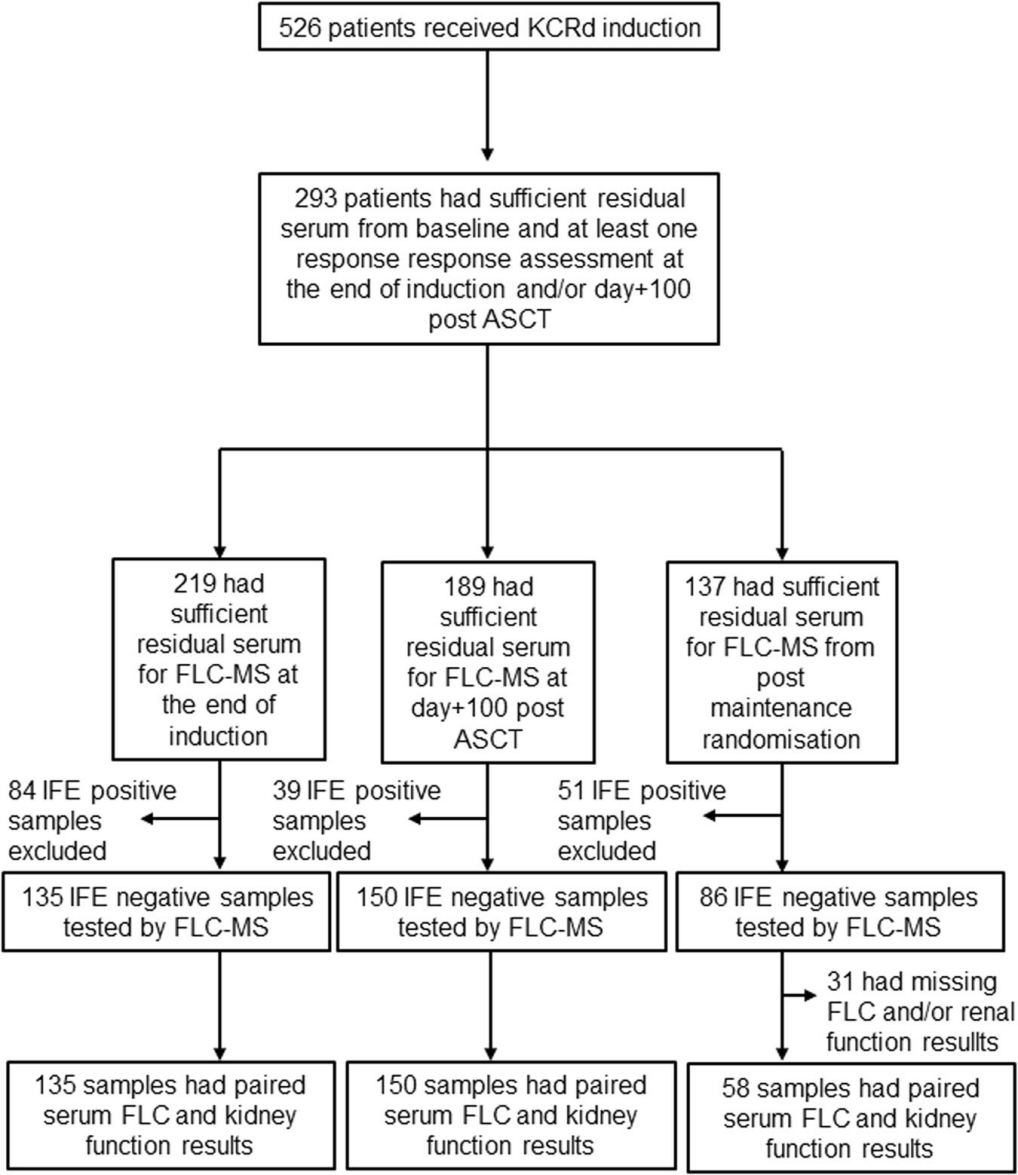
CONSORT diagram. CONSORT diagram showing the flow of patients included in this study.

Centralised response assessments using protein electrophoresis, IFE, serum FLC measurements and flow cytometry-based MRD (minimum sensitivity 4 × 10^−5^) were conducted as part of the trial [22]. All patients with sufficient residual serum for MS testing from baseline sample or the end of cycle one of induction chemotherapy and at least one follow-up sample from within 100 days post-ASCT were included in this study (Fig. 1).

Estimated glomerular filtration results (eGFR) were calculated using the modified CKD-EPI calculation [23]. Patients with eGFR ≥ 30 ml/minute/1.73 m^2^ were categorised as having a persistently abnormal serum FLC ratio if the ratio was <0.26 for patients with lambda light chain secreting disease and >1.65 for patients with kappa light chain secreting disease. Patients with eGFR <30 ml/minute/1.73 m^2^ were categorised as having a persistently abnormal serum FLC ratio if the direction of skew was consistent with the involved light chain component of the monoclonal protein identified at baseline and the FLC ratio was outside the modified renal reference range of 0.37–3.1 [24].

### FLC-MS testing

**Fig. 2:**
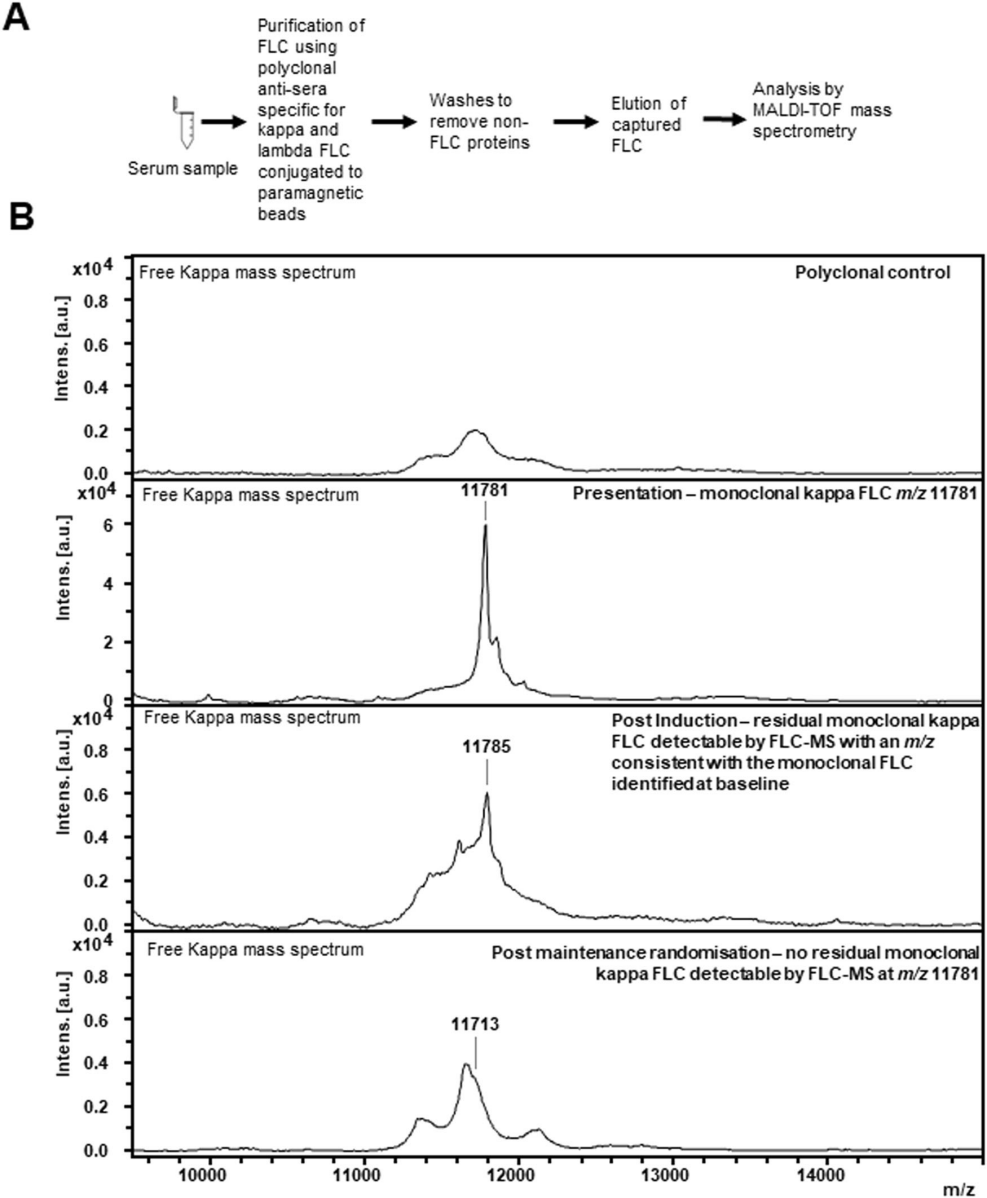
Workflow and example mass spectra showing how FLC-MS is used to identify and track monoclonal FLC across serial samples. The workflow for analysis of a serum sample by FLC-MS is shown in (**A**). **B** shows example mass spectra of how FLC-MS is used to track monoclonal FLC across serial samples. An example mass spectrum from a polyclonal sample run against free kappa is shown in the top mass spectrum. At presentation monoclonal kappa FLC with an m/z of 11781 for the doubly charged light chain were identified. Persistent residual monoclonal kappa FLC are detectable by FLC-MS (peak at m/z 11785 for the doubly charged light chain) at the end of induction chemotherapy. There is also an oligoclonal peak within the postinduction spectrum with a completely distinct m/z to the monoclonal light chain. At the postmaintenance time point there are no residual monoclonal kappa FLC detectable by FLC-MS. There is a very small abnormality in the kappa FLC spectrum (*m/z* 11713 for the doubly charged light chain) which is likely due to a very small oligoclonal peak.

FLC-MS assessments were performed on a BRUKER microflex MALDI-TOF mass spectrometer (Bruker,GmBH, Germany). Serum was prepared for analysis by diluting it 1:4 in phosphate-buffered saline and incubating it with polyclonal FLC-specific antisera (The Binding Site Ltd, Birmingham, United Kingdom) conjugated to magnetic microparticles for thirty minutes. Non-immunoglobulin proteins were removed by washing the samples with phosphate-buffered saline and standard de-ionised water. Captured FLC were eluted using 5% acetic acid and 20 mmol tris(2-carboxyethyl)phosphine (The Binding Site Ltd), spotted onto MALDI-TOF target plates and analysed over a mass range of 5000–32000 Da. Mass spectra were reviewed using FlexAnalysis (Bruker, GmBH, Germany) by trained operators who were blinded to the central laboratory testing results. FLC-MS spectra were interpreted in a binary fashion i.e. they were classified as positive if a peak of the same FLC isotype and a *m/z* consistent with that observed at baseline was present and negative if not (Fig. 2).

### Statistical analysis

Statistical analysis was performed using SPSS version 29.0. Progression-free survival (PFS) was measured from enrolment to progression or death from any cause. PFS results were analysed using the Kaplan-Meier method and statistical significance of results was tested using the log-rank test. Univariate and multivariate analysis were performed using the Cox proportional hazards model and results were considered statistically significant if the *p* value was less than 0.05. Median follow-up duration was calculated using the reverse Kaplan-Meier method.

### Data sharing

The data that support the findings of this study are available from the corresponding author by email request.

## Results

### Patients and disease characteristics

526 patients with transplant-eligible multiple myeloma were randomised to be treated with KCRD induction, followed by a high-dose melphalan conditioned ASCT and a 2:1 randomisation between continuous lenalidomide maintenance versus observation in the Myeloma XI trial. Residual serum from baseline and at least one follow-up sample in which there was no monoclonal protein detectable by IFE were available in: 135 patients from the end of induction chemotherapy; 150 patients from day+100 post-ASCT; and 86 patients from six months post maintenance randomisation. FLC-MS was performed on paired baseline and follow-up samples in all of these patients.

The baseline characteristics of the 222 patients included in this study are listed in Table 1. At presentation 215/220 (96.8%) patients with FLC results available at baseline had an abnormal serum FLC ratio and in 186/220 (83.8%) patients the ratio of involved: uninvolved FLC was >10 at presentation. Overall, 203/222 (91.4%) patients received a high-dose melphalan-conditioned ASCT and 126/222 (56.8%) received continuous lenalidomide maintenance until progression or intolerance. The median follow-up duration for patients included in this study was 42.9 months.

**Table 1:** Baseline Characteristics of the Patients Included in this Study.

	Baseline Characteristics
**Age** < **65 years, n (%)**	161/222 (72.5%)
**Monoclonal protein isotype**	
IgG, n (%)	112/222 (50.5%)
IgA, n (%)	64/222 (28.8%)
FLC only, n (%)	43/222 (19.4%)
Other, n (%)	3/222 (1.4%)
**Monoclonal protein level (g/L), median (range)**	30.5 (0.27–101.7)
**Involved Light Chain Isotype**	
Kappa, n (%)	140/222 (63.1%)
Lambda, n (%)	82/222 (36.9%)
**Abnormal serum FLC ratio**	215/222 (96.8%)
**missing, n (%)**	2/222 (0.9%)
**Serum FLC ratio** > **10**	186/222 (83.8%)
**missing, n (%)**	2/222 (0.9%)
**involved FLC (mg/L), median (range)**	434.9 (0.37–40347)
**Serum creatinine (µmol/L), median (range)**	89 (43–315)
**Received high dose melphalan ASCT**	
**Yes, n (%)**	203/222 (91.4%)
**No, n (%)**	19/222 (8.6%)
**Maintenance randomisation**	
**Lenalidomide, n (%)**	126/222 (56.8%)
**Observationor not randomised, n (%)**	96/222 (43.2%)

### Detection rates for residual monoclonal FLC by freelite and FLC-MS

**Fig. 3:**
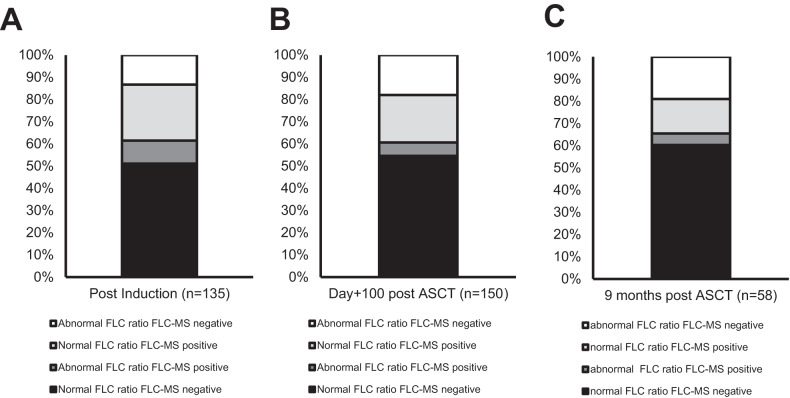
Agreement between serum FLC ratio and FLC-MS assessments. Agreement between serum FLC ratio and FLC-MS assessments at the end of induction chemotherapy (**A**), day+100 post-ASCT (**B**) and 6 months post maintenance randomisation are shown in (**C**).

32/135 (23.7%) of samples from patients in whom there was no detectable monoclonal protein by IFE at the end of induction had an abnormal serum FLC ratio and residual monoclonal FLC was detectable by MS in 47/135 (34.8%) samples. Overall, there was agreement between serum FLC and FLC-MS assessments in 82/135 (60.7%) samples (Fig. 3A).

Most of the discrepant results were due to FLC-MS positivity in samples with a normal FLC ratio. However, there were also 19/53 samples with an abnormal FLC ratio but no residual monoclonal protein detectable by FL-MS. Within these 19 patients, the abnormal FLC ratio was due to suppression of the uninvolved FLC in 13 (68.4%) (supplementary Table 1). 3/6 (50%) of the remaining FLC-MS negative with an abnormal FLC ratio would have been classified as having a normal FLC ratio using the new FLC ratio reference ranges proposed by the iSTOPMM study [25, 26].

**Fig. 4:**
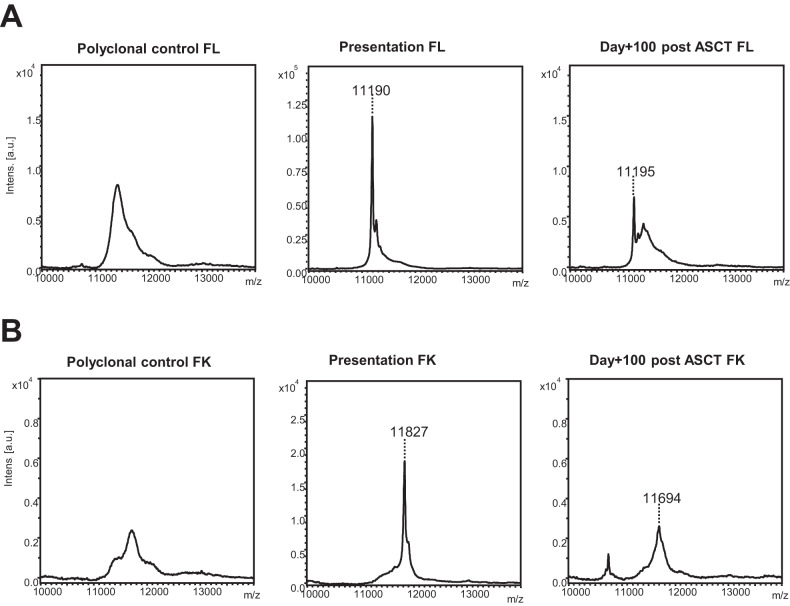
Example mass spectra from patients with discrepant serum FLC ratio and FLC-MS assessments. **A** shows example mass spectra from a patient with a normal serum FLC ratio at day+100 post-ASCT but persistent positivity by MS is shown in. **B** shows example mass spectra from a patient with an abnormal serum FLC ratio at day+100 post-ASCT but no residual monoclonal FLC were identified by FLC-MS. The large oligoclonal peak with a different *m/*z was present and was the likely the cause of the abnormal serum FLC ratio observed in this sample.

At day+100 post ASCT agreement between Freelite and FLC-MS assessments was observed in 91/150 (60.7%) IFE-negative patients with available serum for FLC-MS testing (Fig. 3B). Residual monoclonal FLC was detectable by MS in 32/114 (28.1%) patients with a normal serum FLC ratio. Example mass spectra from a patient with a normal serum FLC ratio but in whom residual monoclonal FLC were detectable by MS are shown in Fig. 4A and example mass spectra from a patient who had an abnormal serum FLC ratio at day+100 but was FLC-MS negative are shown in Fig. 4B.

18/27 (66.7%) patients who were FLC-MS negative but had an abnormal FLC ratio at day +100 post-ASCT would be re-classified as having a normal FLC ratio using the amended iSTOPMM FLC ratio reference ranges [25, 26]. In the remaining nine FLC-MS negative patients with an abnormal FLC ratio at day+100 post-ASCT all of the abnormal FLC ratios were due to suppression of the uninvolved FLC (supplementary Table 1).

Amongst the 86 patients who had no residual monoclonal protein detectable by IFE 58/86 (67.4%) had complete FLC and renal function results available from the postmaintenance randomisation central analysis time point. 43/58 (74.1%) of these patients were randomised to receive lenalidomide maintenance post-ASCT. There was agreement between serum FLC and FLC-MS testing results in 38/58 (65.5%) samples (Fig. 3C). Amongst the 20 samples with conflicting results, 9/20 (45.0%) were FLC-MS positive but had a normal serum FLC ratio. MRD results were available for 6/11 (54.5%) patients with an abnormal serum FLC ratio who were FLC-MS negative and 6/6 (100%) were MRD negative.

In contrast to the patterns observed at the earlier time points, none of the abnormal FLC ratios in FLC-MS negative patients at the post maintenance randomisation time point were due to suppression of the uninvolved FLC. This suggests that at this later time point these discrepant results are more likely to be due to oligoclonal immune reconstitution rather than treatment-related immune suppression.

At all three time points kappa was over-represented as the involved light chain isotype in IFE-negative patients with an abnormal serum FLC ratio. Kappa was the involved light chain in: 22/32 (68.8%), 34/36 (94.4%) and 14/14 (100%) samples from patients with a persistently abnormal serum FLC ratio at post-induction, day+100 post-ASCT and six months post maintenance randomisation respectively. In contrast, no bias towards a specific light chain isotype was observed in patients with persistent positivity by FLC-MS (24/47 (51.1%), 21/41 (51.2%) and 8/18 (44.4%) patients with residual monoclonal FLC detectable by MS at the end of induction chemotherapy, day+100 post-ASCT and six months post maintenance randomisation respectively had kappa secreting disease).

### Residual detection by FLC-MS and serum FLC assessments compared to bone marrow MRD

**Fig. 5:**
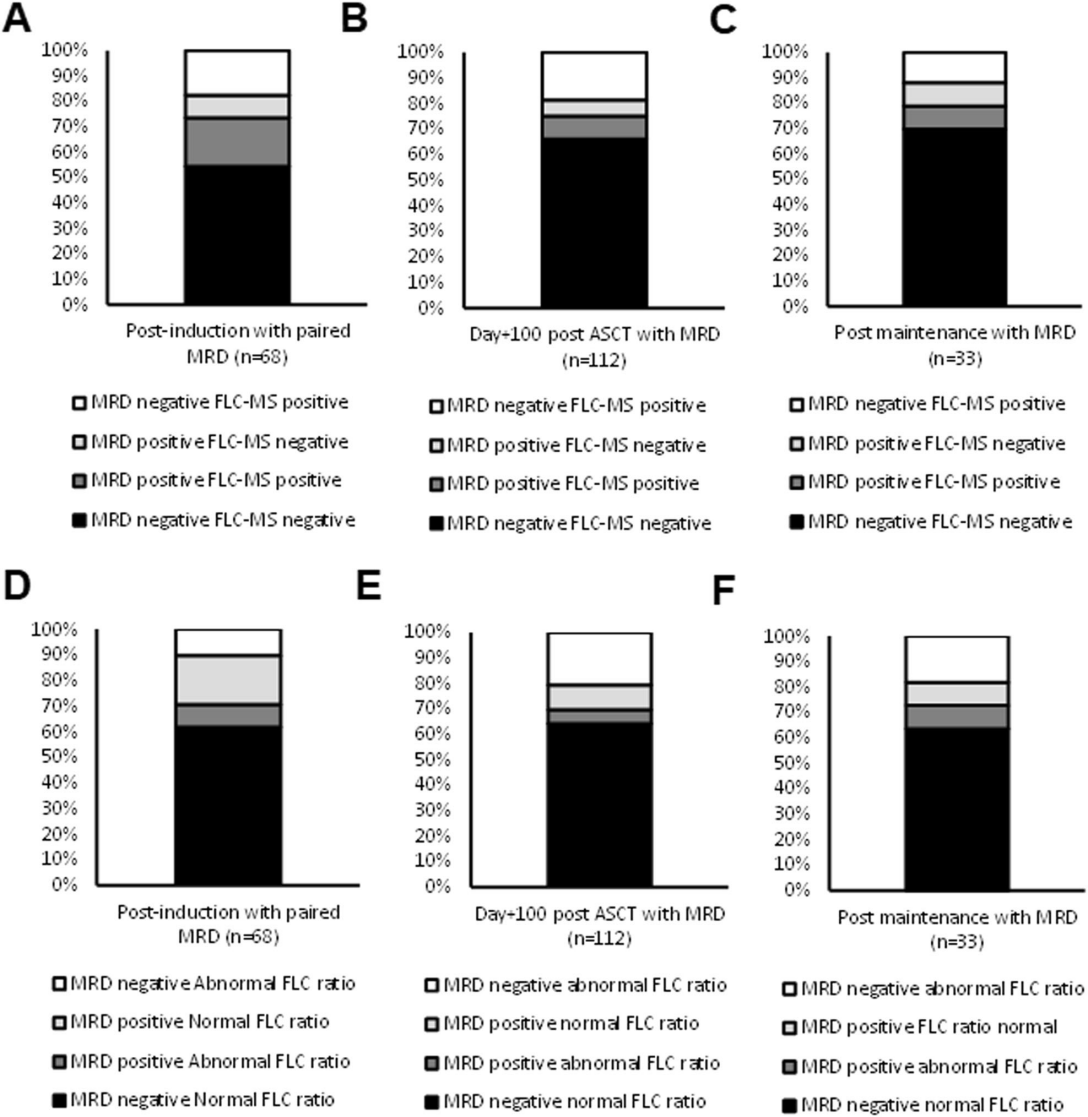
Agreement between serum FLC, FLC-MS and bone marrow MRD assessments. Agreement between bone marrow MRD and FLC-MS assessments in patients at the end of induction chemotherapy (**A**), day+100 post ASCT (**B**) and postmaintenance randomisation (**C**). The agreement between bone marrow MRD and serum FLC assessments at postinduction, day+100 post-ASCT and post maintenance randomisation are shown in (**D–F**).

Concordant results between MRD and FLC-MS assessments were observed in 50/68 (73.5%), 84/112 (75.0%) and 26/33 (78.8%) at the end of induction chemotherapy, day+100 post-ASCT and six months post maintenance randomisation in whom paired results were available (Fig. 5A–C). At all three time points, the majority of discrepant results were due to FLC-MS positivity in the absence of detectable MRD in the bone marrow, which may in part reflect the low sensitivity of the MRD assay included in this trial.

Agreement between bone marrow MRD and serum FLC assessments were observed in 48/68 (70.6%), 78/112 (69.0%) and 24/33 (72.7%) patients at the end of induction chemotherapy, day+100 post-ASCT and six months post maintenance randomisation. At the end of induction the majority of discrepant results were due to MRD positivity in patients with a normal serum FLC ratio (Fig. 5D) and only 1/7 (14.3%) had residual monoclonal FLC by MS. However, at the two post-ASCT timepoints the majority of discrepant results were due to an abnormal serum FLC ratio in the absence of residual detectable MRD in the bone marrow (Fig. 5E, F), which likely reflects the higher rate of oligoclonal immune reconstitution post-ASCT, causing more false positives. This is also supported by the low rate of FLC-MS positivity in these patients (4/23 (17.3%) and 0/6 (0%) had residual monoclonal FLC detectable by MS at day+100 post-ASCT and post maintenance randomisation, respectively).

### Prognostic significance of serum FLC and FLC-MS assessments in IFE-negative patients

**Fig. 6:**
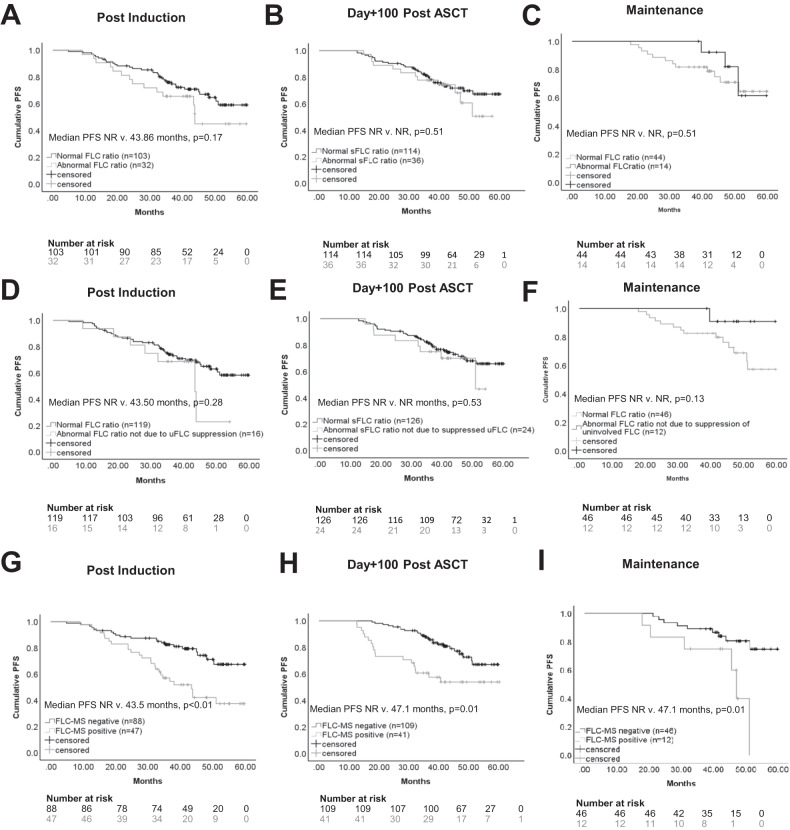
PFS according FLC status at the end of induction chemotherapy, day + 100 post-ASCT and 6 months post maintenance randomisation. **A**–**C** Show the PFS for IFE-negative patients with a normal versus abnormal serum FLC ratio at the end of induction chemotherapy, day+100 post-ASCT and six months post maintenance randomisation. **D**–**F** show the PFS for IFE negative with patients with a normal FLC ratio (classified as a normal serum FLC ratio or an abnormal serum FLC ratio due to suppression of the uninvolved FLC) versus an abnormal FLC ratio at the end of induction chemotherapy, day+100 post-ASCT and six months post maintenance randomisation. **G**–**I** show the PFS for IFE-negative patients according to FLC-MS status at the end of induction chemotherapy, day+100 post-ASCT and six months postmaintenance randomisation.

Normalisation of the serum FLC ratio did not confer any survival benefit in patients with no detectable monoclonal protein by IFE at the end of induction chemotherapy, day+100 post-ASCT or six months post-maintenance randomisation (Fig. 6A–C). This held true even when patients with an abnormal FLC ratio due to suppression of the uninvolved FLC were removed from the abnormal serum FLC ratio group (Fig. 6D–F) and when only patients with an abnormal serum FLC ratio and an elevated level of the involved FLC ratio were considered.

Residual positivity by FLC-MS correlated with reduced PFS at all three-time points (Fig. 6G–I). Even when only patients with intact immunoglobulin-secreting myeloma were included, FLC-MS positivity was still associated with reduced PFS (supplementary Fig. 1). At the end of induction chemotherapy and day+100 post ASCT residual disease detectable by FLC-MS and MRD positivity were both associated with reduced PFS on univariate analysis and retained statistical significance on multivariate analysis (Table 2). FLC-MS and MRD status at the six months postmaintenance time point were also predictive of PFS on univariate analysis. Multivariate analysis including MRD status was not performed at this time point due to the limited numbers of patients with available MRD results. Overall survival was not analysed due to the limited duration of follow-up and limited number of events.

**Table 2:** Univariate and Multivariate Analysis of Factors Affecting PFS.

	n/N	Univariate analysis			Multivariate Analysis 1			Multivariate Analysis 2		
		HR	95% CI	*p* value	HR	95% CI	*p* value	HR	95% CI	*p* value
**Post Induction**										
FLC-MS positive	47/135	2.68	1.51–4.77	<0.001	4.45	1.64–12.02	0.003	4.48	1.65–12.18	0.003
MRD positive	19/68	3.96	1.63–9.59	0.002	2.65	1.05–6.68	0.039	3.24	1.19–9.93	0.022
Age	135/135	1.03	1.00–1.07	0.068				1.04	0.97–1.11	0.28
ISS 2/3	66/133	0.9	0.51–1.60	0.72						
Non-IgG	77/135	1.29	0.72–2.30	0.4						
abnormal sFLC ratio	32/135	1.55	0.83–2.89	0.17						
**Day** + **100 post-ASCT**										
FLC-MS positive	41/150	2.59	1.41–4.75	0.002	2.64	1.30–5.38	0.008	2.44	1.18–5.02	0.016
MRD positive	17/112	2.74	1.27–5.93	0.01	2.33	1.07–5.11	0.034	2.77	1.23–6.24	0.014
Age	150/150	1.03	0.99–1.07	0.08	NI			1.05	1.00–1.10	0.049
ISS 2/3	80/150	1.31	0.71–2.40	0.38	NI			NI		
Non-IgG	69/150	1.04	0.57–1.90	0.91	NI			NI		
abnormal sFLC ratio	36/150	1.254	0.64–2.44	0.51	NI			NI		
**Post Maintenance**										
FLC-MS positive	18/86	3.27	1.33–8.07	0.01	NI			NI		
MRD positive	6/33	4.14	1.01–16.75	0.049	NI			NI		
Age	86/86	1.03	0.98–1.08	0.285	NI			NI		
ISS 2/3	43/85	0.88	0.37–2.07	0.76	NI			NI		
Non-IgG	33/86	1.19	0.50–2.83	0.694	NI			NI		
abnormal sFLC ratio	14/68	0.66	0.18–2.33	0.513	NI			NI		

## Discussion

Several recent studies have recently demonstrated that high throughput MALDI-TOF MS-based assays provide greater sensitivity for the detection of residual monoclonal protein compared to electrophoretic techniques, however, this is the first large clinical study comparing the sensitivity and prognostic implications of a MALDI-TOF MS assay using FLC specific antisera to serum FLC assays such as Freelite. Although a sCR defines the deepest level of response achievable level of response outside of MRD assessments, recent studies have questioned the prognostic significance of FLC ratio normalisation in patients who have achieved a CR [5, 7]. Overall, discordant results between serum FLC and FLC-MS assessments were observed in almost 40% of patients in this study and in keeping with the previous studies normalisation of the serum FLC ratio did not provide any additional prognostic information in IFE-negative patients. However, persistent positivity by FLC MS in IFE-negative patients at the end of induction chemotherapy, day+100 post-ASCT, and six months post maintenance randomisation was associated with reduced PFS.

It has previously been suggested that the lack of prognostic significance of serum FLC ratio normalisation is due to false positives due to treatment-related immune suppression and oligoclonal bands [10, 14, 15]. Oligoclonal immune reconstitution may cause abnormal FLC ratios in the absence of residual detectable monoclonal FLC by FLC-MS as FLC-MS can differentiate between residual disease and an oligoclonal peak even if they are of the same light chain isotype based on the unique *m/z* of the monoclonal FLC identified at baseline. Whilst false positives were an important factor in this study, false negatives due to the limited sensitivity of the current serum FLC assays were also an important factor in this study as residual monoclonal FLC were detectable by MS in 28.7% of samples in which the serum FLC ratio was normal. Importantly, FLC have a short half-life and therefore the detection of residual monoclonal FLC in these patients cannot be attributed to the increased sensitivity of the MS-based assays coupled with long protein half-life.

FLC-MS assays have the advantage that they specifically track the monoclonal FLC rather than relying on skewing of the FLC ratio, which provides indirect evidence of residual disease. MS assays are therefore less susceptible to the effects of treatment-related immune suppression or inflammatory states, which can cause minor skewing of the FLC ratio in the absence of residual disease. The ability of the FLC-MS assay to specifically track the monoclonal light chain is also likely to be particularly useful in patients with renal impairment, where FLC levels and ratios can be particularly challenging to interpret and multiple amended reference ranges have been proposed [24, 25, 27].

Although this study has provided useful insights into the potential utility of FLC-MS for enhancing the sensitivity of serological monitoring for patients undergoing treatment for multiple myeloma it does have some limitations. Firstly, due to the limited duration of follow-up overall survival could not be analysed. Secondly, flow-cytometry results were missing for a large number of the patients and the flow cytometry assay used in this trial was low sensitivity (minimum sensitivity 4 × 10^−5^) compared to that achieved with next-generation sequencing and next-generation flow cytometry MRD assays. Lastly, serum FLC assay interpretation is even more challenging in the context of severe renal impairment where several different adjusted ranges have been proposed [24, 25, 27] and none have been validated or universally adopted. The Myeloma XI trial did not enrol patients with dialysis-dependent renal failure or acute kidney injury with a serum creatinine ≥500µmol/L at presentation, therefore the utility of FLC-MS in this patient cohort could not be evaluated in this study.

At present this assay requires manual inspection of the mass spectra by trained operators to classify the spectra as being positive or negative, which means that throughput has not been maximised. Software that could automatically screen and interpret the mass spectra, similar to that used for the MASS-FIX and QIP-MS/EXENT assays, is currently under development. Once this has been refined and validated automated interpretation of spectra, including quantitative analysis, would be possible on a high throughput basis in a similar manner to that done for the quantitation of intact immunoglobulin monoclonal proteins by the QIP-MS/EXENT assay. This would allow graded responses according to the amount of residual monoclonal FLC detected by mass spectrometry to be assigned and may be particularly important if this assay is to be used in conditions such as AL amyloidosis where the degree of FLC reduction is central to response assessments.

Additionally, whilst alternative and potentially cheaper assays that can provide greater sensitivity for the detection of monoclonal FLC compared to the existing techniques are also under development, they are much more labour-intensive and therefore not currently suitable for high throughput clinical use [28, 29]. If they can be adapted for use in high throughput diagnostic laboratories they should be compared to FLC-MS in future studies to identify the most efficient and cost-effective methodology for enhancing the sensitivity of FLC measurements in the serum.

In conclusion, this study has demonstrated that FLC-MS offers a superior methodology for monitoring monoclonal FLC in the serum compared to current techniques. The detection of residual FLC by MS but not by indirect measures using the serum FLC ratio identifies patients in serological CR at higher risk of early progression. The serum FLC ratio is a less accurate method to assess residual clonal FLC in this patient group and FLC-MS demonstrates that the serum FLC ratio has both a significant false positive and false negative rate. If the findings of this study are validated in other prospective studies, refinement of the current definition of a sCR should be considered.

Further evaluation of FLC-MS is needed in prospective studies of patients undergoing treatment for multiple myeloma and comparison to other methods for monitoring MRD. With the improving depths of responses achieved with modern treatment regimens more sensitive tests are needed to help differentiate between differing depths of response and FLC-MS represents a promising potential addition to the diagnostic armamentarium. The enhanced sensitivity offered by FLC-MS may be particularly beneficial when monitoring patients with plasma cell disorders associated with low-level monoclonal FLC production such as AL amyloidosis and monoclonal gammopathy of renal significance. It may also be particularly valuable in patients with renal failure where serum FLC values and ratios are particularly troublesome to interpret and FLC-MS should therefore also be further explored in this patient cohort in future studies.

### Supplementary information

Supplementary Figure 1.

Supplementary figure 1 legend

Supplementary Table 1.

## Data Availability

The data will be available by email request from hannah.giles@uhb.nhs.uk or guy.pratt@uhb.nhs.uk.
