# Supplementary figures and images for: Progression free survival of myeloma patients who become IFE-negative correlates with the detection of residual monoclonal free light chain (FLC) by mass spectrometry
Source: Blood Cancer J. 2024 Mar 18;14(1):50. doi: 10.1038/s41408-024-00995-y (PMC10948753; doi:10.1038/s41408-024-00995-y)

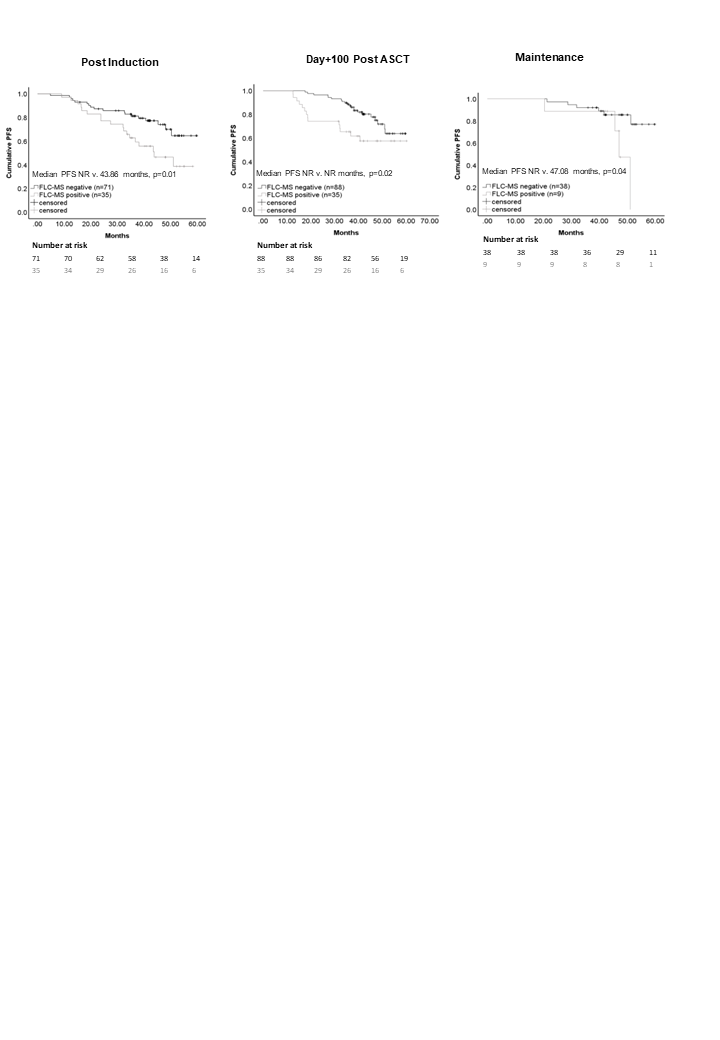

Supplement: Supplementary file 1 — Supplementary Figure 1. [file 41408_2024_995_MOESM1_ESM.tif]
