# Supplementary material for: Progression free survival of myeloma patients who become IFE-negative correlates with the detection of residual monoclonal free light chain (FLC) by mass spectrometry
Source: Blood Cancer J. 2024 Mar 18;14(1):50. doi: 10.1038/s41408-024-00995-y (PMC10948753; doi:10.1038/s41408-024-00995-y)
Supplement: Supplementary file 2 — Supplementary figure 1 legend [file 41408_2024_995_MOESM2_ESM.docx]

**Supplementary Figure 1. PFS according to FLC-MS status at the end of induction chemotherapy, day+100 post ASCT and six months post maintenance randomisation restricted to patients with intact immunoglobulin monoclonal protein secreting myeloma only.**
